# Proportions and trends of critical care trials in leading general medical journals, 1970–2022

**DOI:** 10.1186/s13054-023-04666-5

**Published:** 2023-09-29

**Authors:** Nin-Chieh Hsu, Charles Liao, Chia-Hao Hsu

**Affiliations:** 1 Division of Hospital Medicine, Department of Internal Medicine, Taipei City Hospital Zhongxing Branch, Taipei, Taiwan; 2 College of Medicine, National Taiwan University, Taipei, Taiwan; 3 Division of Hospital Medicine, Department of General Medicine, Stanford University, School of Medicine, Stanford, CA USA; 4 Department of Orthopedics, Kaohsiung Medical University Hospital, Kaohsiung Medical University, No. 100, Shih-Chuan 1st Road, Sanmin Dist., Kaohsiung City, 80708 Taiwan; 5 College of Medicine, Kaohsiung Medical University, Kaohsiung, Taiwan

To the Editor,

The field of critical care is expansive, encompassing a wide range of topics [1, 2] and various comprehensive skills [3]. The current journal alert systems or subscriptions may not offer intensivists sufficient access to comprehensive information. Clinical trials in the field of critical care can certainly be published in top-ranked general medical journals. However, the proportions and trends of publication between journals were never reported. We conducted a cross-sectional meta-research assessment on critical care trials published from 1970 to 2022. We did not seek approval from the institutional review board because the study did not involve human beings and the data were in the public domain.

We examined the quantity and trends of publications in the field of critical care by Medical Subject Headings (MeSH), a controlled vocabulary thesaurus developed by the National Library of Medicine [4]. The MeSH categories do not include major topics specifically related to critical care. To identify relevant studies, we systematically searched PubMed using six specific MeSH terms: “Critical Care”, “Critical Care Nursing”, “Critical Care Outcomes”, “Intensive Care Units”, “Intensive Care Units, Pediatric”, and “Intensive Care, Neonatal”. A similar methodology was employed in a recent report [5]. Furthermore, we restricted the publication type to clinical trial, a predefined category of publication type in the PubMed database, and limited the publication dates from January 1, 1970, to December 31, 2022. A flow chart of trial selection is provided (Additional file 1: Fig. S1).

To analyze publications per year for each general medicine journal, we focused on the New England Journal of Medicine (NEJM), Lancet, Journal of the American Medical Association (JAMA), and British Medical Journal (BMJ). These four journals were selected because they published research across a broad spectrum of topics in the field of medicine and had the leading citations in the category of Medicine, General and Internal according to Journal Citation Reports of 2022. Additionally, all four journals published on a weekly basis which allows a fair comparison of quantity and trend.

We assessed for a linear trend over time by constructing the data for the periods of 1970–1987, 1988–2005, and 2006–2022 using a general linear model. For trend analysis between the journals, we utilized the Cochrane Armitage test. We performed two sensitivity analyses. First, to mitigate the potential influence of the COVID-19 pandemic, we performed a sensitivity analysis by excluding publications from the years 2019–2021. Second, we limited the trend analysis to recent two decades (2003–2022).

A total of 157,655 articles were identified through the MeSH terms related to critical care medicine (as of July 7, 2023). Among them, a total of 7,663 critical care clinical trials conducted between 1970 and 2022 were included. We observed a significant increasing trend in the number of critical care clinical trial publications using a general linear model (*p* for trend < 0.001). Among these trials, 86 (1.12%) were published in NEJM, 54 (0.70%) in Lancet, 114 (1.49%) in JAMA, and 24 (0.31%) in BMJ. The combined publications of the top four leading general medical journals comprised 3.63% of all critical care trials. The annual publication trends in JAMA increased when referenced by Lancet (*p* for trend < 0.001) (Fig. 1). This remained consistent after excluding the COVID-19 pandemic years 2019–2021 (Additional file 2: Table S1). Notably, in the recent two decades (2003–2022), statistically significant publication trends emerged between JAMA and BMJ (Additional file 2: Table S1).

**Fig. 1:**
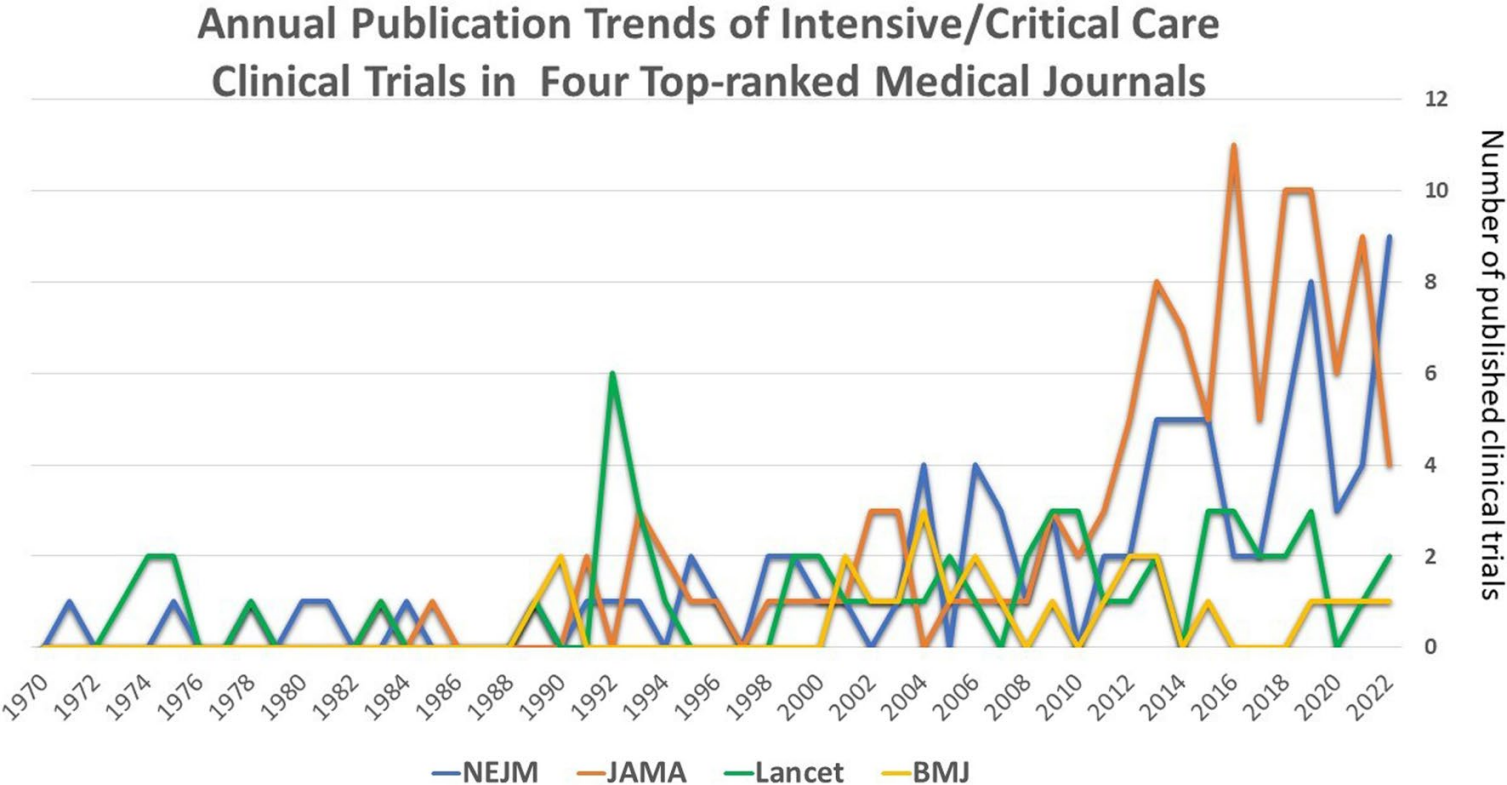
The annual trend in the number of published clinical trials between 1970 and 2022 was examined in four leading general medical journals. The results revealed a significant increase in the number of published trials for NEJM and JAMA, whereas no significant change was observed for Lancet and BMJ over this period (Cochrane Armitage test, *p* for trend < 0.001). In the most recent decade (2013–2022), JAMA published a total of 75 trials, whereas BMJ published only 7 trials

Our analyses revealed distinct patterns in publication quantity and trends among top-ranked general medical journals in the field of critical care. The NEJM and JAMA published a higher number of clinical trials in critical care and exhibited similar increasing publication trends. In contrast, the Lancet and BMJ published fewer critical care trials without clear trends. Our study had certain limitations. First, there was an uncontrolled tendency for authors of these trials to submit their work to specific journals, which may have introduced bias. Second, the evolution of impact factors of the journals may have confounded the results. However, the quantity of publications was not in concordance with the impact factors. Third, the definition and classification of clinical trials may vary across different scientific databases, leading to potential issues of mislabeling and bias. As a result, the findings presented in this study are confined to the PubMed database, and the emphasis should be placed on identifying trends rather than simply assessing publication quantity. Finally, it is important to note that different editorial policies may exist among these journals. These findings highlight the varying areas of interest among these leading general medical journals. Consequently, intensivists may receive unequal exposure to the latest knowledge of critical care depending on their subscription or alert preferences from different journals.

### Supplementary Information

**Additional file 1: Figure S1.** Flow chart of stepwise selection of critical care clinical trials in the PubMed Database using pre-defined MeSH terms.

**Additional file 2: Table S1.** Results of Cochrane Armitage test for trends between four leading general medical journals.

## Data Availability

The datasets used and/or analyzed during the current study are available from the corresponding author on reasonable request.

## Abbreviations

BMJ: British Medical Journal
JAMA: Journal of the American Medical Association
MeSH: Medical Subject Headings
NEJM: New England Journal of Medicine
