# Supplementary figures and images for: Proportions and trends of critical care trials in leading general medical journals, 1970–2022
Source: Crit Care. 2023 Sep 29;27:375. doi: 10.1186/s13054-023-04666-5 (PMC10540386; doi:10.1186/s13054-023-04666-5)

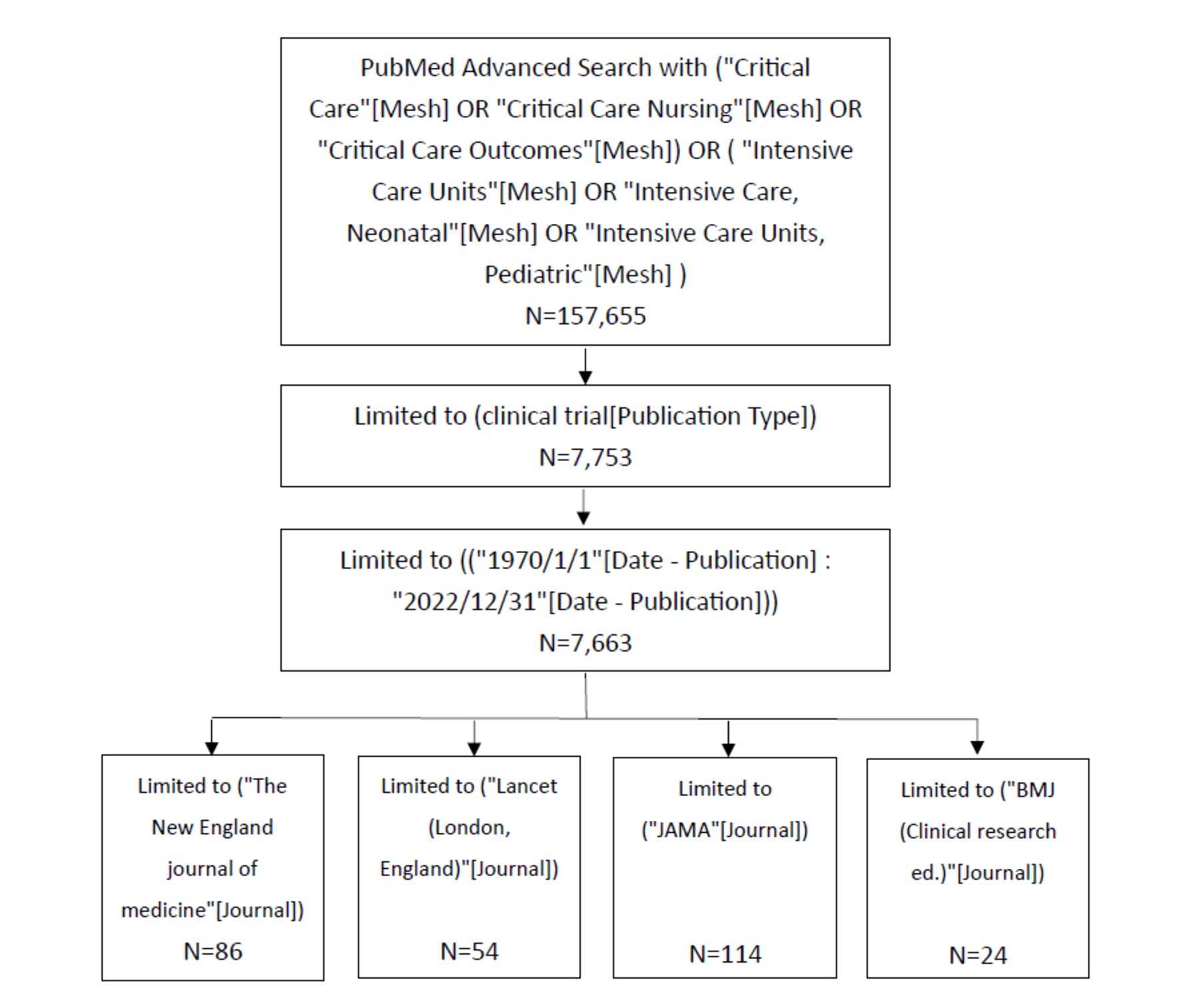

Supplement: Supplementary file 1 — Additional file 1: Figure S1. Flow chart of stepwise selection of critical care clinical trials in the PubMed Database using pre-defined MeSH terms. [file 13054_2023_4666_MOESM1_ESM.jpg]
