# Supplementary material for: Proportions and trends of critical care trials in leading general medical journals, 1970–2022
Source: Crit Care. 2023 Sep 29;27:375. doi: 10.1186/s13054-023-04666-5 (PMC10540386; doi:10.1186/s13054-023-04666-5)
Supplement: Supplementary file 2 — Additional file 2: Table S1. Results of Cochrane Armitage test for trends between four leading general medical journals. [file 13054_2023_4666_MOESM2_ESM.docx]

| **Table S1**. Results of Cochrane Armitage test for trends between journals | | | |
| --- | --- | --- | --- |
| **Journals** | **P value for trend** | | |
|  | **1970-2022** | **1970-2018, 2022*** | **2003-2022** |
| NEJM vs JAMA | 0.1716 | 0.1732 | 0.5273 |
| NEJM vs Lancet | 0.0575 | 0.1271 | 0.3624 |
| NEJM vs BMJ | 0.5576 | 0.6807 | 0.1075 |
| JAMA vs Lancet | <0.0001 | 0.0033 | 0.1184 |
| JAMA vs BMJ | 0.0053 | 0.1213 | 0.0235 |
| Lancet vs BMJ | 0.4190 | 0.4754 | 0.4653 |

Definition of abbreviations: BMJ=British Medical Journal; NEJM=New England Journal of Medicine; JAMA=Journal of the American Medical Association.

*Exclude COVID years 2019-2021
